# Co-Expression Network Analysis Identifies miRNA–mRNA Networks Potentially Regulating Milk Traits and Blood Metabolites

**DOI:** 10.3390/ijms19092500

**Published:** 2018-08-24

**Authors:** Adolf A. Ammah, Duy N. Do, Nathalie Bissonnette, Nicolas Gévry, Eveline M. Ibeagha-Awemu

**Affiliations:** 1 Agriculture and Agri-Food Canada, Sherbrooke Research and Development Centre, 2000 College Street, Sherbrooke, QC J1M 0C8, Canada; Achu.Ammah.Adolf@USherbrooke.ca (A.A.A.); DuyNgoc.Do@AGR.GC.CA (D.N.D.); Nathalie.Bissonnette@AGR.GC.CA (N.B.); 2 Department of Biology, University of Sherbrooke, 2500 University Boulevard, Sherbrooke, QC J1K 2R1, Canada; Nicolas.Gevry@USherbrooke.ca; 3 Department of Animal Science, McGill University, 21111 Lakeshore Road, Ste. Anne de Bellevue, QC H9X 3V9, Canada

**Keywords:** co-expression, microRNA, mRNA, transcription factor, blood metabolites, milk components, milk fatty acids

## Abstract

MicroRNAs (miRNA) regulate mRNA networks to coordinate cellular functions. In this study, we constructed gene co-expression networks to detect miRNA modules (clusters of miRNAs with similar expression patterns) and miRNA–mRNA pairs associated with blood (triacylglyceride and nonesterified fatty acids) and milk (milk yield, fat, protein, and lactose) components and milk fatty acid traits following dietary supplementation of cows’ diets with 5% linseed oil (LSO) (*n* = 6 cows) or 5% safflower oil (SFO) (*n* = 6 cows) for 28 days. Using miRNA transcriptome data from mammary tissues of cows for co-expression network analysis, we identified three consensus modules: blue, brown, and turquoise, composed of 70, 34, and 86 miRNA members, respectively. The hub miRNAs (miRNAs with the most connections with other miRNAs) were miR-30d, miR-484 and miR-16b for blue, brown, and turquoise modules, respectively. Cell cycle arrest, and p53 signaling and transforming growth factor–beta (TGF-β) signaling pathways were the common gene ontology (GO) and Kyoto Encyclopedia of Genes and Genomes (KEGG) pathways enriched for target genes of the three modules. Protein percent (*p* = 0.03) correlated with the turquoise module in LSO treatment while protein yield (*p* = 0.003) and milk yield (*p* = 7 × 10^−04^) correlated with the turquoise model, protein and milk yields and lactose percent (*p* < 0.05) correlated with the blue module and fat percent (*p* = 0.04) correlated with the brown module in SFO treatment. Several fatty acids correlated (*p* < 0.05) with the blue (CLA:9,11) and brown (C4:0, C12:0, C22:0, C18:1n9c and CLA:10,12) modules in LSO treatment and with the turquoise (C14:0, C18:3n3 and CLA:9,11), blue (C14:0 and C23:0) and brown (C6:0, C16:0, C22:0, C22:6n3 and CLA:10,12) modules in SFO treatment. Correlation of miRNA and mRNA data from the same animals identified the following miRNA–mRNA pairs: miR-183/*RHBDD2* (*p* = 0.003), miR-484/*EIF1AD* (*p* = 0.011) and miR-130a/*SBSPON* (*p* = 0.004) with lowest *p*-values for the blue, brown, and turquoise modules, respectively. Milk yield, protein yield, and protein percentage correlated (*p* < 0.05) with 28, 31 and 5 miRNA–mRNA pairs, respectively. Our results suggest that, the blue, brown, and turquoise modules miRNAs, hub miRNAs, miRNA–mRNA networks, cell cycle arrest GO term, p53 signaling and TGF-β signaling pathways have considerable influence on milk and blood phenotypes following dietary supplementation of dairy cows’ diets with 5% LSO or 5% SFO.

## 1. Introduction

Bovine milk and its products constitute a rich source of proteins, energy, minerals (e.g., calcium), vitamins (A, B, D, E and K) and antioxidants in human nutrition. Milk supplies unsaturated fatty acids (USFA) which have been associated with decreased risk of cardiovascular diseases (stroke, high blood pressure, heart failure and coronary heart diseases), inflammatory diseases and some types of cancers [1,2,3]. Unsaturated fatty acids make up about 30% of the fatty acid content of milk, meanwhile it has been proposed that milk fat composition with potential positive effects on human health should contain about 70% USFA [4]. Nutrition is one of the factors that greatly impacts milk fat composition and the largest changes in milk fatty acid composition have been obtained either by changing the amounts and the nature of forages in the diets of cows, particularly pasture, or by adding plant or marine oils to the diet [5,6]. Plant products like linseed, soybeans, safflower and sunflower are the most effective sources of unsaturated plant lipids used to enhance the conjugated linoleic acid (CLA) and USFA contents of milk fat. Unsaturated fatty acids and other factors like physiological and metabolic state of the cow, breed and genetics are known to influence the concentration of blood metabolites like glucose, nonesterified fatty acids (NEFA), triacylglyceride (TAG) and β hydroxybutyric acid [7,8]. For instance, we reported significant increases in blood NEFA and TAG concentrations and significant reductions in milk fat and milk urea nitrogen contents in Holstein cows following dietary supplementation with USFA [8]. Moreover, the blood metabolic profile of dairy cows is used to assess the nutritional and health state of the dairy herd [9,10].

In our previous transcriptome studies of the bovine mammary gland, we identified mRNAs and miRNAs that were differentially expressed in response to diets rich in USFA [11,12]. Furthermore, we also examined the effect of diets rich in USFA on milk composition (fat, protein, milk yield and lactose) and blood metabolites (TAG and NEFA) of lactating Holstein cows [7]. The mRNA transcriptome analysis identified 1006 (460 up and 546 downregulated) and 199 (127 up and 72 down-regulated) genes that were significantly differentially regulated after linseed oil (LSO) and safflower oil (SFO) supplementation, respectively, meanwhile the miRNA transcriptome analysis detected 14 and 22 miRNAs significantly differentially regulated by LSO and SFO, respectively. Since a network of genes and regulatory factors work in concert to influence the phenotypic expression of traits, assessment of gene expression without taking into account the factors that regulate their activities may not adequately explain the complex biological mechanisms underlying the expression of traits. MiRNAs interact with mRNA(s) to regulate their (mRNA(s)) expression and consequently biological processes, so it is important to study their synergistic effects on the phenotypic expression of traits. Hence, an integrative approach in assessing gene expression in a network basis is necessary to unravel the molecular mechanism underlying milk fat traits.

Network approaches have proven to be powerful tools for exploring the biological mechanisms underlying complex traits [13,14,15]. It has been widely applied on output data from high throughput sequencing technology to identify key regulators and pathways in human diseases such as cancer and obesity [16], type 1 diabetes [17], Alzheimer’s disease [18], livestock production traits [13,19,20,21], and functional annotation of cattle genes [22]. Moreover, understanding gene networks also helps to better guide genomic selection in animal breeding programs [23]. In order to understand gene interaction, different methods have been developed to construct co-expression networks and to identify modules of highly connected genes. The weighted gene co-expression network analysis (WGCNA) is among the most established and widely used of such methods [24]. We and other authors have successfully used WGCNA to identify key genes and networks for various complex traits in livestock species such as meat quality traits in pigs [25], feed efficiency in cattle [26] and milk yield and component traits in cows [27]. Furthermore, integrative omics approaches have been applied on combined mRNA and miRNA expression data to detected major regulatory mechanisms in different phenotypes such as carcass and meat quality traits in porcine [25], abnormality in breast cancer patients [28], and colorectal cancer [29]. Moreover, the consensus module approach (finding common functions/processes) has proven to be a promising method for finding hub genes and regulators across different datasets [30,31,32,33,34,35]. Such hub genes and regulators may form targets for further functional validation of their roles in identified networks. Furthermore, the identification of key miRNAs, their networks, and their downstream target genes and pathways might also facilitate the use of genetic engineering technologies, such as RNA interference technologies or gene editing, to obtain desired phenotypes by controlling the expression of miRNAs and/or their target genes. Moreover, the miRNAs, genes, and network information might be useful for genomic predictions [36,37]. However, these approaches have not yet been applied to explore the regulatory mechanisms in the bovine mammary gland in response to diets rich in USFA (SFO or LSO). Therefore, this study aimed to (i) construct consensus modules across miRNA expression data from control, LSO and SFO treatments using the WGCNA approach; (ii) correlate important miRNA modules with milk and blood component phenotypes; (iii) enrich target genes (mRNA) of miRNAs from important modules to explore the possible biological processes, pathways and transcriptional regulators regulating milk and blood component phenotypes and (iv) identify miRNA–mRNA networks regulating milk and blood component phenotypes.

## 2. Results

### 2.1. Phenotypic Data

A summary of the data on blood metabolites, milk and component yields including fatty acid profiles for the control and treatment periods used for co-expression and network analyses is shown in Table 1.

### 2.2. Identification of Consensus Modules and Module Trait Relationship

**Figure 1:**
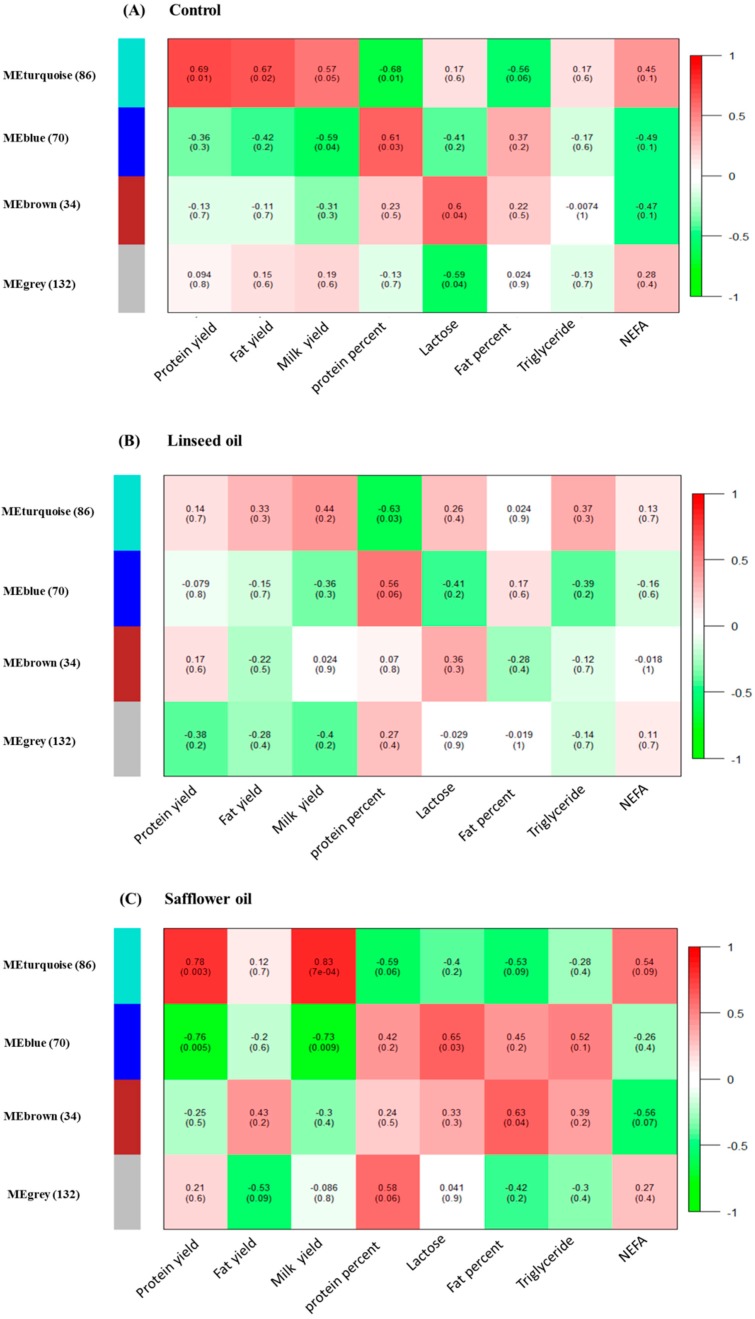
Consensus modules and module-trait relationship matrix: The weighted gene co-expression network analysis (WGCNA) was used to group miRNAs into consensus modules based on their expression patterns. Four consensus modules were identified and each consensus module eigengene was tested for correlation with blood and milk parameters during (**A**) control period, (**B**) linseed oil and (**C**) safflower oil treatments. Correlation coefficients and corresponding *p*-values (in brackets) between turquoise, blue and brown modules in the *y*-axis, and blood and milk parameters in the *x*-axis. The module−trait relationship matrix is colored based on the intensity of the correlation: red is a strong positive correlation, while green is a strong negative correlation.

**Figure 2:**
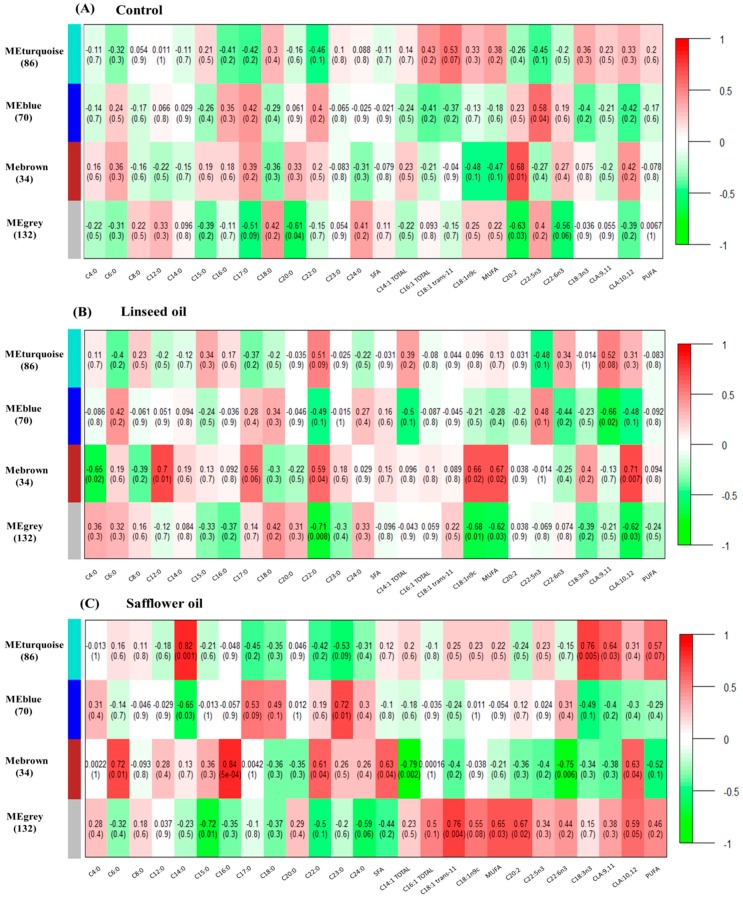
Consensus module−trait relationship matrix: The weighted gene co-expression network analysis (WGCNA) was used to group miRNAs into consensus modules based on their expression patterns. Four consensus modules were identified and each consensus module eigengene was tested for correlation with milk fatty acid traits during (**A**) control period, (**B**) linseed oil and (**C**) safflower oil treatments. Correlation coefficients and corresponding p-values (in brackets) between miRNA modules in the *y*-axis and milk fatty acids in the *x*-axis. The module−trait relationship matrix is colored based on the intensity of the correlation: red is a strong positive correlation, while green is a strong negative correlation.

Using the WGCNA approach for miRNA read data described by Li et al. [12], we identified a total of three consensus modules (blue, brown, and turquoise) of co-expressed miRNAs during the control and treatment periods (Figure 1 and Figure 2). The modules were made up of 70 (blue), 34 (brown), and 86 (turquoise) miRNA members (Figure 1 and Figure 2). The grey module grouped miRNAs with no coherent co-expression patterns; therefore it was not further discussed. MiRNAs were further selected based on their intra modular connectivity or eigengene-based connectivity (k.ME). The k.ME is a measure of how a miRNA is correlated to module eigengene and miRNAs with high k.ME values (>0.6) are better representatives of module characteristics [38]. Therefore, miRNAs with k.ME > 0.6 were selected for downstream analyses. A total of 18, 12 and 19 miRNAs with k.ME > 0.6 in the blue, brown, and turquoise modules, respectively, were selected for downstream analyses (Table 2). Hub miRNAs or miRNAs with the most connections with other members of the module were bta-miR-30d, bta-miR-484 and bta-miR-16b for the blue, brown, and turquoise modules, respectively (Table 2).

In LSO treatment, the turquoise module correlated significantly with protein percent (*p* = 0.03) while protein yield (*p* = 0.003) and milk yield (*p* = 7 × 10^−4^) correlated with the turquoise module, protein and milk yields and lactose percent (*p* < 0.05) correlated with the blue module and fat percent (*p* = 0.04) correlated with the brown module in SFO treatment (Figure 1). Several fatty acids correlated (*p* < 0.05) with the blue (CLA:9,11) and brown (C4:0, C12:0, C22:0, C18:1n9c and CLA:10,12) modules in LSO treatment and with the turquoise (C14:0, C18:3n3 and CLA:9,11), blue (C14:0 and C23:0) and brown (C6:0, C16:0, C22:0, C22:6n3 and CLA:10,12) modules in SFO treatment (Figure 2). Several measured parameters also correlated with the identified modules in the control samples (Figure 1 and Figure 2).

### 2.3. miRNA Target Gene Prediction and Enrichment Analysis

Using TargetScan, we identified 3199, 3727, and 4045 target mRNAs for 18, 12, and 19 miRNAs in the blue, brown, and turquoise modules, respectively (Table S1a,c,e). Amongst them, 1311, 1533, and 1697 mRNAs from mRNA data of the same samples [11] had significant negative correlations (FDR < 0.05) with 18, 12 and 19 miRNAs in the blue, brown, and turquoise modules, respectively (Table S1b,d,e). These mRNAs (filtered target mRNAs) were used as input for enrichment analyses for GO, Kyoto Encyclopedia of Genes and Genomes (KEGG) pathways and transcription factors.

**Figure 3:**
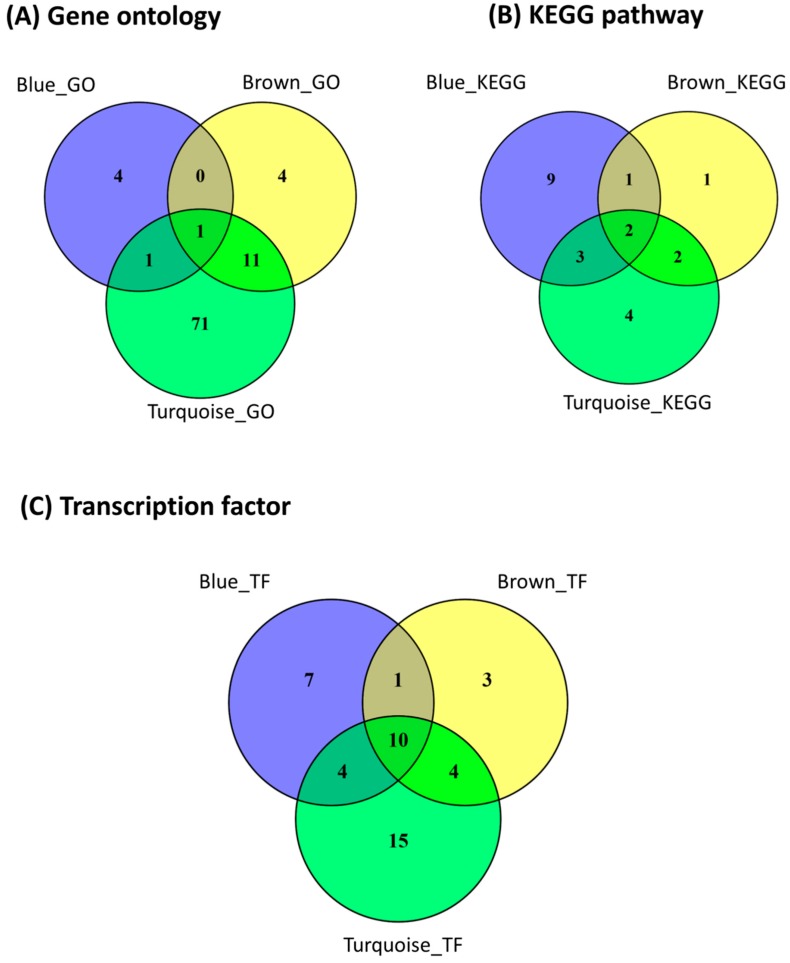
Venn diagrams showing number of enriched unique and shared (**A**) gene ontology terms, (**B**) Kyoto encyclopedia of genes and genomes (KEGG) pathways and (**C**) transcription factors for predicted target genes of miRNA in the blue, brown, and turquoise modules. GO: Gene ontology, KEGG; KEGG pathway, TF: Transcription factor.

A total of 6, 16, and 84 GO terms were enriched for filtered target mRNAs of the blue, brown, and turquoise modules, respectively (Table 3). The GO term, cell cycle arrest (GO: 0007050) was common to the three modules (Figure 3, Table 3). Vesicle docking (GO: 0048278), GDP binding (GO: 0019003) and GTP binding (GO: 0005525) were the most significantly enriched GO terms for the blue, brown, and turquoise modules, respectively (Table 3).

A total of 15, 6, and 11 KEGG pathways were enriched for the blue, brown, and turquoise modules, respectively (Table 4). Two KEGG pathways (p53 signaling and transforming growth factor (TGF) β signaling pathways) were common to the three modules (Table 4, Figure 3). Also, five (p53 signaling, cell cycle, Forkhead box O (FoxO) signaling, protein processing in endoplasmic reticulum and TGF-β signaling pathways) and four (TGF-β signaling, endocytosis, p53 signaling and ubiquitin mediated proteolysis pathways) pathways were common to the blue and turquoise, and brown and turquoise modules, respectively (Figure 3, Table 4). The most significantly enriched pathways in the blue, brown, and turquoise modules were p53 signaling pathway (*p* = 9.93 × 10^−4^), mitogen-activated protein kinase (MAPK) signaling pathway (*p* = 3.40 × 10^−2^) and ubiquitin mediated proteolysis pathway (*p* = 5.81 × 10^−7^), respectively.

A total of 22, 18 and 33 transcription factors were enriched for the blue, brown, and turquoise modules (*p* < 0.05), respectively (Figure 3, Table 5). Ten transcription factors (*SMAD4, SP1, EGR1, NRF1*, *STAT3*, *E2F1*, *TP53*, *MEF2A*, *ATF4*, and *HIF1A*) were common to the three modules. Also, four (*NFYA*, *LEF1*, *PCBP1* and *ATF2*) transcription factors were common to the blue and turquoise modules and another four (*PLAU*, *THRB*, *E2F6* and *TEAD4*) were common to the brown and turquoise modules. *SMAD4* was the most significantly enriched transcription factor for the blue (*p* = 1.28 × 10^−7^) and turquoise (*p* = 3.85 × 10^−11^) modules; meanwhile, *SP1* was the most significantly enriched (*p* = 2.41 × 10^−8^) transcription factor for the brown module.

### 2.4. Integration of miRNA–mRNA and Trait Relationship

**Figure 4:**
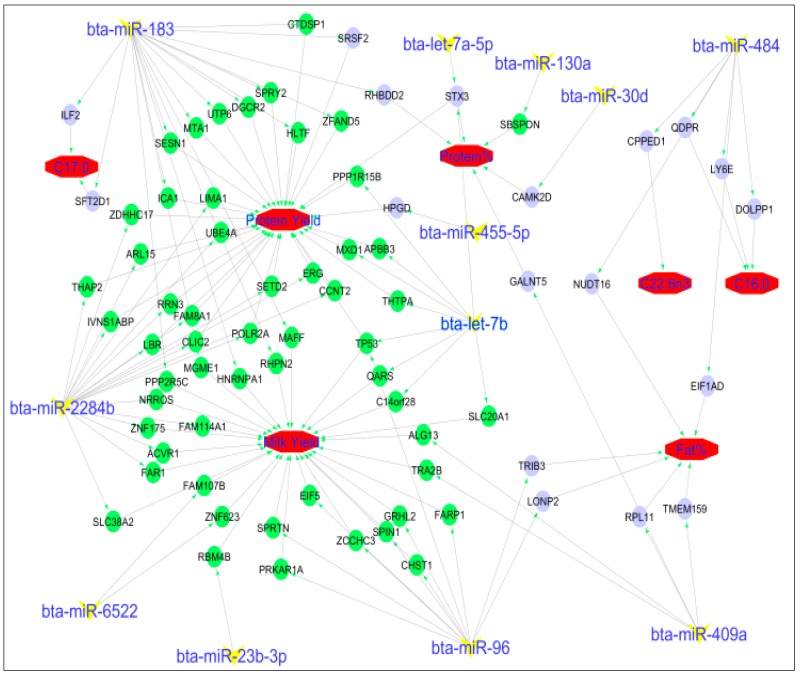
miRNA–mRNA-trait relationships. The mRNAs (green circles (negative correlation) or blue circles [positive correlation]) were significantly correlated with traits (octagonal red shapes). MiRNA (‘Y’ yellow shapes) were significantly correlated with traits (positively or negatively) and negatively correlated with corresponding mRNAs.

A total of 132 of 1311 filtered target mRNAs, 19 of 1533 filtered target mRNAs and 2 of 1697 filtered target mRNAs were negatively and significantly correlated with miRNAs in the blue, brown, and turquoise modules, respectively (Table S1g). The most significantly correlated miRNA–mRNA pairs were bta-miR-183/*RHBDD2* (*p* = 0.003), bta-miR-484/*ADM2* (*p* = 0.001) and bta-miR-130a/*SBSPON* (*p* = 0.004) in the blue, brown, and turquoise modules, respectively. Moreover, 68, 6 and 2 miRNA–mRNA pairs were found to be significantly correlated with at least one phenotype at FDR < 0.05 (Table 6, Figure 4). Milk yield, protein yield, and protein percentage were correlated (FDR < 0.05) with 28, 31 and 5 miRNA–mRNA pairs, respectively (Table 6, Figure 4).

## 3. Discussion

Previously, we reported effects of diets rich in USFA on milk components and blood metabolites and on mRNA and miRNA expression in the bovine mammary gland [7,11,12]. In the current study, we performed WGCNA of the miRNA data of the same animals and identified miRNA consensus modules (blue, brown, and turquoise) as well as miRNA–mRNA co-expressed pairs with potential effects on milk components, fatty acid phenotypes and blood metabolites. Nine miRNA members of the blue (bta-miR-30d, miR-96, miR-409a, miR-183, miR-99a-5p, miR-2285k, miR-652, miR-6522 and miR-374a), 5 of the brown (bta-let-7d, miR-885, miR-29b, miR-32 and miR-107) and 14 of the turquoise (bta-miR-16b, miR-130a, miR-142-5p, miR-218, miR-142-3p, miR-195, miR-19b, miR-455-3p, miR-15a, miR-424-5p, miR-106b, miR-155, miR-93 and miR-99a-3p) modules were previously reported as differentially expressed between lactation stages [39]. Additionally, 5 miRNAs (bta-miR-30d, miR-191, miR-151-5p, miR-99a-5p and let-7d), two miRNAs (bta-miR-26b and let-7g) and one miRNA (bta-miR-142-5p) in the blue, brown, and turquoise modules, respectively, were most abundant in milk fat, milk whey, milk cell and mammary gland tissues of lactating Holstein cows [40] further supporting the influence of the blue, brown, and turquoise miRNA members on milk yield and components in this study. Previous studies have demonstrated the effects of diets rich in USFA on milk and blood components. Salehi et al. [41] demonstrated the effects of diets rich in USFA on blood NEFA levels in dairy cows. Furthermore, increased blood plasma levels of NEFA in cows fed diets supplemented with flaxseed (linseed) and fish oil have been reported [42,43,44]. Similarly, TAG concentrations in serum increased when cows in early lactation received increasing levels of dietary fatty acids from canola oil (1% canola oil +1% fish oil, or 2% canola oil) [45].

### 3.1. Blue Module miRNAs and Their Potential Roles

The blue module miRNAs were significantly correlated with milk and protein yields during the control period, milk yield, protein percent and lactose during treatment with SFO, protein percent and protein yield with LSO treatment. The miRNA expression data of these animals revealed that several of the blue module miRNAs were differentially expressed in response to dietary supplementation with SFO (bta-miR-96, miR-99a-5p, miR-199b, miR-16a, miR-484 and miR-99b) and LSO (bta-miR-885, miR-23b-3p and miR-99a-5p) [12], thus supporting their relationship to milk yield and milk components.

Many of the blue module miRNAs have been reported to play diverse roles in many biological processes. For example, human homologue of bta-miR-191 has been found to be dysregulated in different types of tumors in humans including colorectal [46], breast and prostate cancers [47], while miR-199b is involved in acute myeloid leukemia [48,49] and breast cancer metastasis [50,51,52]. The implication of these miRNAs (bta-miR-191 and miR-199b) in the different types of cancers suggests roles in cellular functions in the mammary gland. The development of breast and prostate cancers is linked to cellular processes like cell death or apoptosis, therefore suggesting a link between these miRNAs and the milk phenotypes in this study.

Some blue module miRNAs like bta-miR-183 and bta-miR-2284b correlated negatively with many candidate genes for protein yield (*CTDSP1*, *DGCR2*, *HLTF*, *ICA1*, *MTA1*, *SESN1*, *SPRY2*, *SRSF2*, *UTP6* and *ZFAND5)*, milk yield (*C14orf28*, *QARS*, *SLC20A1*, *HNRNPA1*, *MAFF*, *MGME1*, *PPP2R5C* and *RHPN2*) and C17:0 fatty acid (*ILF2* and *SFT2D1*) (Figure 4) thus suggesting potential roles in the regulation of these genes. In addition to the report of differential expression of some blue module miRNAs between lactation stages [39], the importance of blue module miRNAs for milk yield and components is further supported by results of pathways enrichments of their target mRNAs. For instance, ErbB, MAPK, Wnt, TGF β and Hippo signaling pathways are involved in the regulation of mammary gland development and lactation processes (reviewed in [53]). Furthermore, ErbB and TGF β signaling pathways have been associated with lactation persistency in Holsteins [54]. The p53 signaling pathway, the second most enriched pathway for the blue module, functions by preventing cancer formation and hence acts as a tumor suppressor [55]. The p53 gene has been nicknamed “guardian of the genome” due to its role in maintaining the stability of the genome [56]. In addition, this pathway regulates proper cellular differentiation and development, and it is also important for tissues undergoing postnatal development [57]. Furthermore, inappropriate expression of the p53 signaling pathway within the mammary epithelium of transgenic mice caused apoptotic cell death of the alveolar epithelium of the mammary gland [58]. In this study, enrichment of p53 signaling pathway supports the significant correlation of the blue module miRNAs with milk yield, protein yield and lactose percent.

Moreover, we also reported several enriched GO terms for the blue module filtered target mRNAs such as vesicle docking, negative regulation of transcription from RNA polymerase II promoter and proteasome−mediated ubiquitin-dependent protein catabolic process. However, it is not clear how these GO terms are linked to the studied phenotypes. We identified 22 important transcription factors which could mediate the functions of miRNAs in the regulation of the target mRNAs or phenotypes (Table 5). *SMAD4*, *SP1* and *EGR1* were the top three transcription factors enriched for the target mRNAs of the blue module miRNAs. *SMAD4* is a tumor suppressor gene and it is essential for transforming growth factor beta (TGFβ) signaling [59], and plays important roles in cell differentiation, growth and apoptosis [60]. Other important transcription factors such as *STAT3* and *PPARG* are well known to regulate milk and milk fat synthesis [61,62,63].

### 3.2. Brown Module miRNAs and Their Potential Roles

Bta-miR-484, a member of the brown module was previously reported as differentially expressed due to LSO and SFO treatments and it is also important in the regulation of lactation signaling [40]. Correlation analyses indicated that this miRNA negatively correlated with previously reported candidate genes for fat percentage (*EIF1AD* and *NUDT16*), C22:6n3 (*CPPED1*) and C16:0 (*LY6E*, *DOLPP1* and *QDPR*). The effects of LSO and SFO supplementation reduced fat percentage in the studied animals by 30.38% and 32.42%, respectively [11] thus supporting the present observation. Additionally, bta-miR-484 has been observed to prevent cell proliferation and epithelial–mesenchymal transition process by targeting both *ZEB1* and *SMAD2* genes, thus functions like a tumor suppressor and may serve as a prospective biomarker for cervical cancer [64]. Other members (bta-miR-26b and bta-miR-107) of the brown module have functions related to cellular processes [40,53] which are essential for lactation processes or milk synthesis. The human homologue of bta-miR-26b was shown to play a protective role in the etiology of breast cancer by promoting apoptosis through targeting *SLC7A11* [65] while bta-miR-107 is associated with mammary stem cell activities [66].

The most significantly enriched biological process GO term in the brown and turquoise modules, GDP binding, is involved in cell proliferation, signal transduction, protein synthesis, and protein targeting [67] (Table 3). Other enriched pathways in the brown module like MAPK signaling pathway plays an important part in numerous cellular processes such as apoptosis, proliferation and differentiation [68], stress responses, and immune defense [69,70] and have been noted to be important for mammary gland development and milk secretion in caprine [71].

The most enriched transcription factor for the brown module was specific protein 1 (*SP1*) known to regulate the expression of numerous genes involved in cell proliferation, apoptosis and differentiation, and increase in its transcriptional activities is associated with tumorigenesis [72].

### 3.3. Turquoise Module miRNAs and Their Potential Roles

The hub miRNA (bta-miR-16a) for the turquoise module has been reported to be differentially expressed in response to SFO treatment [12]. Bta-miR-16a being differentially regulated by SFO and also having the highest intra modular connectivity suggests involvement in the regulation of the traits (C14:0, C18:3n3 and 9, 11-CLA) that were significantly correlated with the turquoise module in SFO treatment. Although a direct role for this miRNA in mammary gland functions has not been demonstrated, a previous study suggests its involvement in tumor suppression through inhibition of cell cycle progression [73]. Some turquoise module miRNAs like bta-miR-130a and bta-miR-142-5p have been linked to milk fat synthesis [40,74] and disease parthenogenesis [75,76,77]. Additionally, overexpression of bta-miR-130a affects cellular triacylglyceride synthesis in bovine mammary epithelial cells via regulation of *PPAR-γ* [74], fatty acid storage and glucose metabolism. Besides, some predicted target genes of miR-130a have been associated with neurodevelopmental disorders such as autism, schizophrenia and hereditary spastic paraplegia [78]. GO enrichment indicated that target mRNAs of turquoise module miRNAs participate in many different processes such as GTP binding, GDP binding, transcription coactivator activity, membrane organization, protein ubiquitination and regulation of apoptotic processes. Several KEGG pathways such as Ubiquitin mediated proteolysis, TGF-β signaling pathway and cell cycle, and transcription factors such as *STAT3*, *SMAD4*, *SP1* and *EGR1* with roles in many different processes involving milk production and related traits [39,63] were enriched for target mRNAs of turquoise module miRNAs. TGF-β signaling pathway and p53 signaling pathway were common pathways enriched by target mRNAs from all three modules and their roles in related traits have been discussed above. Ubiquitin mediated proteolysis, the most enriched pathway for turquoise module is important for protein degradation [77] and many processes including mediation of lactation signal in skeletal muscle of dairy cows [76]. Top transcription factors enriched for the target mRNAs of turquoise module were *SMAD4*, *SP1*, and *EGR1*.The early growth response protein 1 (*EGR1*) acts as a transcription regulator of target genes, hence play roles in the regulation of cell survival, proliferation, cell death response to growth factors, DNA damage, and ischemia [79]. A notable enriched transcription factor was *STAT3* with known association with milk production [75], fertilization and embryonic survival rates [80] in dairy cows.

### 3.4. Association Between miRNA and mRNA with Expressed Phenotypes

The most interesting part of this analysis is the different pathways linking miRNAs to the actual phenotypes through their target genes (Table 6 and Figure 4). Interestingly, different miRNAs that shared the same target genes regulated different traits. For instance, by targeting *TP53*, bta-let-7b might influence milk yield but also, bta-miR-96 might have an influence on protein percentage. Since many genes (mRNAs) and miRNAs influencing milk yield have been characterized such as *TP53*, *GRHL2,* bta-miR-183, bta-let-7b and bta-miR-96, we will not discuss about the network of miRNAs influencing milk yield. Meanwhile, for the first time, the link between some milk fatty acids, genes (mRNAs) and miRNAs have been reported. Interestingly, we observed negative correlation between bta-miR-484 and three different genes (*QDPR*, *LY6E* and *DOLPP1*) and positive correlation with C16:0 concentrations in milk. The roles of bta-miR-484 have been reported above, while it is not clear how these three genes are involved in the metabolism of C16:0. However, *DOLPP1* has a potential role in regulating subcutaneous fat [81] while *LY6E* might play a role in the regulation of glycosylphosphatidylinositol. Also, *QDPR* is important for fat traits in pigs [82]. Therefore, these genes might be interesting candidates for milk fat traits. The influence of bta-miR-183 on protein yield and protein percentage suggests that it can down regulate 8 different genes to influence protein yield. Nevertheless, these connections are based on correlations so it might not reflect true associations; therefore more studies are needed to validate the identified links and how they participate in mammary gland response to dietary USFA.

## 4. Materials and Methods

### 4.1. Animal Management and Sampling

Animal management and sampling procedures were according to the national codes of practice for the care and handling of dairy cows (http://www.nfacc.ca/codes-of-practice) and approved by the Animal Care and Ethics Committee of Agriculture and Agri-Food Canada (CIPA#402, 04 April 2012).

Detailed procedures for animal management and data collection have been reported previously [12]. Briefly, 12 Canadian Holstein cows in mid-lactation were randomly assigned to either LSO or SFO treatment (6 cows/treatment). These animals were fed a control diet of total mixed ration of corn and grass silages (50:50) and concentrates for 28 days (control period), after which the control diet was supplemented with 5% LSO or 5% SFO on a dry matter basis (treatment period) for another 28 days. Animals were milked every day at 8:00 am and 6:00 pm. Analysis of fat, protein and lactose contents in milk samples collected on days −14 and −1 (control period) and +7, +14, +21 and +28 (treatment period) were done using 80 mL of milk (pool of morning (40 mL) and evening (40 mL) milk) by a commercial laboratory (Valacta Laboratories Inc., Ste. Anne de Bellevue, QC, Canada). Daily milk yield for each cow was recorded with electronic milk meters (MU-480, De Laval Inc., Kansas City, MO, USA). Milk fat from milk samples (40 mL) was extracted by centrifugation at 4500× *g* for 20 min at 4 °C.

Blood samples were aseptically collected from animals on days −14 and −1 (control period) and +7, +14, +21 and +28 (treatment period) and centrifuged at 7500× *g* for 20 min at room temperature. The resulting plasma was used for the analysis of non-esterified fatty acid (Wako Chemicals, Kit HR series NEFA-HR, Richmond, VA, USA) and triacylglyceride (enzychrom TAG assay kit, Bioassay System, Hayward, CA, USA), following manufacturers’ instructions.

Mammary biopsies were collected from animals in each group on day −14, day +7 and day +28 which corresponded to middle of control period, early treatment and end of treatment periods, respectively, following an established protocol [83]. Biopsies were snap frozen in liquid nitrogen and stored at −80 °C pending isolation of total RNA.

### 4.2. RNA Isolation

Procedures for RNA isolation have been reported previously [11]. In brief, 50 mg of mammary gland biopsy sample was used for total RNA isolation using miRNeasy Kit (Qiagen Inc., Toronto, ON, Canada) following manufacturer’s instructions. The Turbo DNase Kit (Ambion Inc., Foster City, CA, USA) was used to remove contaminating DNA from isolated RNA. The Nanodrop ND-1000 instrument (NanoDrop Technologies, Wilmington, DE, USA) was used to measure RNA concentration and Agilent 2100 Bioanalyzer (Agilent Technologies, Santa Clara, CA, USA) was used to accessed RNA quality. The RNA Integrity Numbers of all the samples were >8.

### 4.3. mRNA Sequencing and Data Processing

Procedures for mRNA sequencing and data processing have been reported previously [11]. Briefly, 250 ng of total RNA/sample was use for library generation using the TruSeq stranded mRNA Sample Preparation Kit (Illumina Inc., San Diego, CA, USA). The Quant-iT™ PicoGreen^®^ dsDNA Assay Kit (Life Technologies, Burlington, ON, Canada) and the Kapa Illumina GA with the Revised Primers-SYBR Fast Universal Kit (D-Mark Biosciences, Toronto, ON, Canada) were used to quatify prepared libraries. The 2100 Bioanalyzer instrument (Agilent Technologies) was used to determine the average fragment size of libraries. Generated libraries (36) were multiplexed and subjected to 100 bp paired-end sequencing on six lanes of a HiSeq 2000 system (Illumina Inc.) by McGill University and Genome Quebec Innovation Centre (Montreal, QC, Canada). Generated reads were processed using a pipeline developed by McGill University and Genome Quebec Innovation Centre (http://gqinnovationcenter.com/).

### 4.4. miRNA Sequencing and Data Processing

Procedures for miRNA library preparing, sequencing and bioinformatics management of data have been reported previously [12]. Briefly, the procedure for miRNA library preparation and barcoding for sequencing was according to Vigneault, et al. [84] with slight modifications [12]. Total RNA was first ligated to a primer (adaptor) at the 3′ by T4 RNA Ligase 22tr K227Q (New England Biolabs Inc., Canada) and 5′ ends by T4 RNA Ligase 1 (Enzymatics Inc., a division of Qiagene Inc., Beverly, MA USA) followed by reverse transcription into cDNA using Superscript III Kit (Life Technologies, Carlsbad, CA, USA). Barcoding of the different libraries was done followed by size separation by polyacrylamide gel electrophoresis and finally the concentration of the purified libraries was assessed by PicoGreen assay (Life Technologies, USA) on a Nanodrop 3300 fluorescent spectrophotometer. Multiplexed libraries were sequenced on 3 lanes on an Illumina HiSeq 2000 system (Illumina Inc., USA) by McGill University and Genome Quebec Innovation Centre (Montreal, QC, Canada).

The FastQC program version 0.10.1 (http://www.bioinformatics.babraham.ac.uk/projects/fastqc/) was used to check sequencing quality. Then, the cutadapt v1.2.2 program (http://code.google.com/p/cutadapt/) was used to trim adaptor sequences. Clean reads were parsed into one and mapped to the bovine genome (Bta_4.6.1) using bowtie 1.0.0. [85]. Reads that mapped to 1 to 5 positions were further used. miRDeep2 v2.0.0.5 tool was used to identify known miRNAs and also in the discovery of novel miRNAs.

### 4.5. Fatty Acid Analysis

Fatty acid methyl ester preparation and quantification of fatty acid profiles have been reported previously [11]. Briefly, fatty acid methyl esters were prepared and quantified using the Hewlett Packard 6890 N gas chromatographic system (Agilent Technology, Wilmington, DE, USA). The carrier gas was hydrogen and the capillary column used was SLB-IL111 (100 m × 0.25 mm, 0.2 µm in thickness, Supelco, Bellefonte, PA, USA). The oven temperature was set at 40 °C for 1 min followed by 80 °C to 170 °C for 1 min, then 40 °C to 195 °C for 2 min and finally 20 °C to 210 °C for 15 min. Individual fatty acids were determined by comparing their retention time with that of fatty acid methyl esters standards (GLC No. 463 and No. UC-59-M, Nu-Chek Prep Inc., Elysian, MN, USA). The Chemstation B.04.03 software (Agilent technologies) was used for data analysis.

### 4.6. Construction of Gene Co-Expression Networks

The expression of miRNAs was normalized using Deseq2 package (v1.11.19) [86] and the final normalized matrix of 321 miRNAs were used as input for co-expression network analysis using the WGCNA R-package [24]. For WGCNA analysis, a signed co-expression measure for each pair of miRNAs was computed based on their co-expression level. Then weighted adjacency matrix was calculated from the signed co-expression measure using a power function. A topological overlap measure (TOM) was then calculated based on a combination of a value between the adjacency of two miRNAs and the connection strength that the two miRNAs share with other miRNAs. A TOM of 0 or 1 was assigned to each pair of miRNAs. When miRNAs shared the same neighbor, a TOM value of 1 is assigned while a TOM value of 0 indicates that they do not share any neighbor. To produce a clustering tree (dendrogram), the dynamic tree−cutting algorithm was used [87]. To construct the consensus module, the blockwiseConsensusModulesfunction was run [88,89] with option of soft-thresholding power for network construction of 9, and minimum module size of 20. Moreover, the medium threshold was also applied to control the sensitivity of module detection (deepSplit of 2) and to merge modules in the dynamic tree (mergeCutHeight of 0.25). The signed network option was chosen when constructing the consensus modules. In the gene network, a gene might interact with many others to perform its function, therefore, a minimum module size of 30 genes has been recommended for the gene network construction, by the software developers [24]. Since a lower number of miRNAs might interact with each other to form networks [90,91], we applied a lower threshold of 20 miRNAs for minimum module size. Each branch of a tree is a module and a module with at least 20 miRNAs was assigned to a color (Figure S1). Details about WGCNA and its merits have been reported previously [24,89,92].

### 4.7. Module−Trait Relationship

Module−trait relationships were computed based on Pearson’s correlation between the module eigengene and blood and milk components data. The eigengene is defined as the first principal component of a given module and it represents a measure of miRNA expression profiles in the module. A module was chosen for further analysis if it presented a module−trait relationship  > |0.5| and a *p*-value < 0.05. Potential biologically interesting (significant) modules were selected for downstream analysis. Furthermore, miRNAs in selected modules were used for functional enrichment analysis if eigengene−based connectivity (k.ME), a measure of how the miRNA is correlated to module eigengene, was > 0.6 (k.ME > 0.6) [26]. A k.ME > 0.6 indicates higher connectivity and thus higher representation of modular functions. 

### 4.8. Predicted Target mRNAs of miRNAs

In order to investigate the function of miRNAs in module significantly correlated with traits, we first predicted their target mRNAs. The perl scripts from the TargetScan website (http://targetscan.org) were used to predict target mRNAs (targetscan_60.pl) and also to calculate their context scores (targetscan_61_context_scores.pl). TargetScan computes the context++ score for a specific site as the sum of the contribution of 14 features of the miRNA, miRNA site, or mRNA (including the mRNA surrounding sequence) (http://www.targetscan.org/vert_70/docs/context_score.html) to define sites on mRNAs most effectively targeted by miRNAs [93]. Predicted target mRNAs with context ++ scores above 95th percentile were further used [12,27,39]. The predicted target mRNAs were then filtered against the mRNA expression data from the same animals [11]. Only target mRNAs that were present in the mRNA expression data were retained for further analysis.

### 4.9. Co-Expression Analysis of miRNA–mRNA Expression

For miRNA–mRNA co-expression, the Pearson correlation coefficient between target mRNAs and miRNAs were calculated. A miRNA–mRNA pair was considered co-expressed if it had a negative and significant correlation value at FDR < 0.05. To further explore how miRNAs contributed to particular traits, we examined the correlation between miRNAs and their mRNA targets with the phenotypes in each significantly correlated module. Important interactions between miRNA and mRNAs were visualized using Cytoscape [94].

### 4.10. Gene Ontologies, Pathways and Transcription Factors Enrichment

Functional enrichment of GO terms of target mRNAs was performed for each selected module using EnrichR [95,96]. EnrichR presents results according to hierarchy and relationship between terms which facilitates the interpretation of results. In this enrichment, the p-values for each term were adjusted using Benjamini–Hochberg (BH) correction [95]. Gene ontology terms, Kyoto Encyclopedia of Genes and Genomes (KEGG) pathways and transcription factors were considered significantly enriched at adjusted *p* < 0.05 (FDR < 0.05).

## 5. Conclusions

In this study, three consensus modules (blue, brown, and turquoise) composed of 70 (blue), 34 (brown) and 86 (turquoise) miRNA members were identified. We also demonstrated how miRNAs in these modules interacted with mRNAs to influence blood and milk phenotypes following dietary supplementation with USFA. Hub miRNAs for the blue, brown, and turquoise modules were bta-miR-30d, bta-miR-484 and bta-miR-16b, respectively. Turquoise module had the most significant correlations with several traits including protein percentage in LSO treatment, protein yield, milk yield, C14:0, C18:3n3n and 9, 11-CLA in SFO treatment. The association of miRNA modules with milk and blood phenotypes has provided information about miRNA modules, hub miRNAs, GO terms, transcription factors and pathways that are involved in the regulation of blood and milk parameters following dietary supplementation with diets rich in USFA. This study will contribute to the molecular understandings of the co-expression patterns of miRNAs, miRNA–mRNA, and regulatory activities in the bovine mammary gland following dietary supplementation with USFA.

## Figures and Tables

**Table 1:** Means (± standard error) of phenotypic data * used for co-expression network analyses.

Trait Acronym	Name	Unit	Control			Linseed Oil Treatment			Safflower Oil Treatment		
			Mean ± SE	Min	Max	Mean ± SE	Min	Max	Mean ± SE	Min	Max
PROT_Y	Protein yield	kg	1.2 ± 0.06	0.99	1.52	1.24 ± 0.06	0.92	1.53	1.17 ± 0.06	0.89	1.47
FAT_Y	Fat yield	kg	1.31 ± 0.08	1.12	1.74	1.07 ± 0.11	0.61	1.98	0.97 ± 0.07	0.67	1.35
Milk	Milk yield	kg	36.94 ± 2.13	29.24	52.24	38.14 ± 2.42	26.44	53.56	36.64 ± 2.86	26.16	53.34
PRT	Protein percentage	%	3.3 ± 0.1	2.9	3.65	3.31 ± 0.11	2.81	4.17	3.27 ± 0.1	2.74	3.85
LAC	Lactose percentage	%	4.7 ± 0.05	4.47	4.88	4.74 ± 0.05	4.43	4.94	4.65 ± 0.04	4.42	4.84
FAT	Fat percentage	%	3.6 ± 0.12	3.07	4.19	2.8 ± 0.19	1.87	3.7	2.77 ± 0.24	1.42	3.99
TAG	Triacylglyceride	nmol/L	0.05 ± 0	0.02	0.07	0.08 ± 0.01	0.04	0.11	0.08 ± 0.01	0.05	0.12
NEFA	Nonesterified fatty acids	nmol/L	103.86 ± 23.55	48.66	312.99	156.48 ± 13.29	92.75	248.53	154.47 ± 14.72	101.13	276.18
C4:0	Butyric acid	mg/100g of fat	2.81 ± 0.35	1.51	4.95	0.64 ± 0.1	0.06	0.95	0.74 ± 0.08	0.03	0.94
C6:0	Caproic acid	mg/100g of fat	2.39 ± 0.18	1.31	3.58	0.97 ± 0.15	0.28	1.51	1.24 ± 0.16	0.52	2.26
C8:0	Caprylic acid	mg/100g of fat	1.18 ± 0.07	1.05	1.66	0.72 ± 0.06	0.5	0.94	0.76 ± 0.05	0.51	1.04
C12:0	Lauric acid	mg/100g of fat	2.77 ± 0.3	1.01	4.68	1.5 ± 0.24	0.56	3.03	1.44 ± 0.21	0.5	2.93
C14:0	Myristic acid	mg/100g of fat	11.43 ± 0.27	10.22	12.7	7.95 ± 0.41	5.47	9.28	6.96 ± 0.51	5.01	9.86
C15:0	Pentadecylic acid	mg/100g of fat	1.44 ± 0.07	1.13	1.86	0.98 ± 0.07	0.68	1.4	0.99 ± 0.05	0.8	1.4
C16:0	Palmitic acid	mg/100g of fat	26.79 ± 0.28	25.46	28.55	21.17 ± 1.2	16.64	28.7	21.94 ± 0.98	17.22	26.7
C17:0	Margaric acid	mg/100g of fat	0.83 ± 0.1	0.44	1.73	0.93 ± 0.12	0.36	1.49	0.73 ± 0.1	0.16	1.29
C18:0	Stearic acid	mg/100g of fat	11.13 ± 0.54	8.02	13.87	8.58 ± 0.49	5.02	10.41	7.57 ± 0.37	5.5	9.02
C20:0	Arachidic acid	mg/100g of fat	0.22 ± 0.03	0.1	0.34	0.21 ± 0.02	0.12	0.29	0.21 ± 0.02	0.1	0.35
C22:0	Behenic acid	mg/100g of fat	0.05 ± 0	0.02	0.07	0.04 ± 0	0.02	0.05	0.04 ± 0	0.03	0.05
C23:0	Tricosanoic acid	mg/100g of fat	0.04 ± 0	0.02	0.05	0.03 ± 0	0.01	0.06	0.04 ± 0	0.02	0.05
C24:0	Lignoceric acid	mg/100g of fat	0.04 ± 0	0.03	0.05	0.03 ± 0	0.01	0.04	0.03 ± 0	0.01	0.05
C14:1total	Myristoleic acid	mg/100g of fat	11 ± 1.13	0.28	1.2	10 ± 1.6	0.25	1.02	11 ± 1.79	0.46	0.98
C16:1total	Palmitoleic acid	mg/100g of fat	1.31 ± 0.06	1.1	1.71	1.65 ± 0.23	0.42	3.08	1.82 ± 0.18	1.21	2.84
C18:1n9c	Oleic acid	mg/100g of fat	19.06 ± 1.35	10.7	24	25.91 ± 1.12	20.08	33.2	22.57 ± 0.7	19.4	26
C20:2	Eicosadienoic acid	mg/100g of fat	0.03 ± 0	0.02	0.05	0.07 ± 0.01	0.01	0.14	0.08 ± 0.01	0.04	0.13
C22:5n3	Docosapentaenoic acid	mg/100g of fat	0.06 ± 0.01	0.02	0.16	0.23 ± 0.04	0.04	0.47	0.14 ± 0.03	0.03	0.32
C22:6n3	Docosahexaenoic acid	mg/100g of fat	0.14 ± 0	0.12	0.16	0.18 ± 0.01	0.13	0.23	0.18 ± 0.01	0.12	0.26
C18:3n3n	α linolenic acid	mg/100g of fat	0.27 ± 0.02	0.2	0.42	0.32 ± 0.02	0.19	0.4	0.21 ± 0.03	0.1	0.49
CLA:9,11	*Cis*-9, *trans*-11 CLA	mg/100g of fat	0.3 ± 0.02	0.16	0.41	0.33 ± 0.02	0.22	0.39	0.31 ± 0.04	0.15	0.54
CLA:10,12	*Trans*-10, *cis*-12 CLA	mg/100g of fat	0.02 ± 0	0.01	0.03	0.04 ± 0	0.03	0.07	0.04 ± 0	0.02	0.06
MUFA	Sum of Monounsaturated fatty acids	mg/100g of fat	22.99 ± 1.38	15.18	28.33	30.59 ± 1.17	24.53	37.83	27.55 ± 0.9	24.32	32.59
SFA	Sum of saturated fatty acids	mg/100g of fat	61.12 ± 0.96	57.42	66.05	43.73 ± 1.49	36.32	51.9	42.7 ± 1.49	35.61	49.1
PUFA	Sum of Polyunsaturated fatty acids	mg/100g of fat	0.84 ± 0.03	0.63	0.99	1.17 ± 0.07	0.82	1.52	0.96 ± 0.1	0.57	1.63

* Data are the means of data collected on days −14 and −1 for the control period and days +7, +14, +21 and +28 for the treatment period. This table presents an overview of the effects of treatments on traits. Detailed results on the effects of treatments on data collected at the different time points have been described previously [7,11].

**Table 2:** Consensus modules (blue, brown, and turquoise) and their miRNA members.

Module	miRNA	^1^ k.ME_ All	*p*-Value k.ME_ All	^2^ k.ME_ Control	*p*-Value k.ME Control	^3^ k.ME_ Linseed	*p*-Value k.ME_ Linseed	^4^ k.ME_ Safflower	*p*-Value k.ME_ Safflower
Blue	bta-miR-30d	0.93	1.58 × 10^−22^	0.96	5.06 × 10^−7^	0.95	1.24 × 10^−6^	0.93	1.69 × 10^−5^
Blue	bta-miR-96	0.89	1.62 × 10^−15^	0.89	6.36 × 10^−5^	0.90	2.62 × 10^−5^	0.90	6.64 × 10^−5^
Blue	bta-miR-191	0.87	4.00 × 10^−20^	0.93	5.88 × 10^−6^	0.97	9.66 × 10^−8^	0.87	2.89 × 10^−4^
Blue	bta-miR-151-5p	0.83	1.04 × 10^−14^	0.90	2.68 × 10^−5^	0.92	1.50 × 10^−5^	0.83	7.43 × 10^−4^
Blue	bta-miR-409a	0.80	6.90 × 10^−12^	0.85	2.47 × 10^−4^	0.80	9.23 × 10^−4^	0.89	1.10 × 10^−4^
Blue	bta-miR-183	0.77	4.79 × 10^−13^	0.90	4.18 × 10^−5^	0.77	1.79 × 10^−3^	0.91	5.11 × 10^−5^
Blue	bta-miR-99a-5p	0.77	1.49 × 10^−14^	0.91	1.82 × 10^−5^	0.77	1.79 × 10^−3^	0.94	1.20 × 10^−5^
Blue	bta-let-7b	0.76	1.66 × 10^−9^	0.76	2.24 × 10^−3^	0.80	8.20 × 10^−4^	0.84	6.75 × 10^−4^
Blue	bta-miR-2285k	0.75	2.89 × 10^−9^	0.75	2.29 × 10^−3^	0.75	2.71 × 10^−3^	0.86	2.96 × 10^−4^
Blue	bta-miR-652	0.73	1.21 × 10^−11^	0.90	2.88 × 10^−5^	0.86	1.59 × 10^−4^	0.73	5.76 × 10^−3^
Blue	bta-let-7a-5p	0.70	9.32 × 10^−9^	0.81	7.38 × 10^−4^	0.81	6.60 × 10^−4^	0.70	8.10 × 10^−3^
Blue	bta-miR-6522	0.68	3.17 × 10^−8^	0.74	2.95 × 10^−3^	0.84	2.87 × 10^−4^	0.68	1.12 × 10^−2^
Blue	bta-miR-100	0.68	2.69 × 10^−8^	0.77	1.57 × 10^−3^	0.68	7.88 × 10^−3^	0.83	7.85 × 10^−4^
Blue	bta-miR-374a	0.66	8.19 × 10^−8^	0.77	1.72 × 10^−3^	0.66	9.28 × 10^−3^	0.80	1.46 × 10^−3^
Blue	bta-miR-2284b	0.66	2.57 × 10^−7^	0.66	9.38 × 10^−3^	0.76	2.06 × 10^−3^	0.76	3.07 × 10^−3^
Blue	bta-miR-532	0.65	1.39 × 10^−7^	0.83	4.06 × 10^−4^	0.65	1.05 × 10^−2^	0.71	7.23 × 10^−3^
Blue	bta-miR-99b	0.64	1.01 × 10^−10^	0.89	4.73 × 10^−5^	0.64	1.28 × 10^−2^	0.88	1.92 × 10^−4^
Blue	bta-miR-23b-3p	0.62	2.65 × 10^−7^	0.77	1.59 × 10^−3^	0.62	1.66 × 10^−2^	0.78	2.15 × 10^−3^
Brown	bta-miR-484	0.78	1.28 × 10^−11^	0.86	1.77 × 10^−4^	0.78	1.27 × 10^−3^	0.88	1.68 × 10^−4^
Brown	bta-let-7d	0.76	1.24 × 10^−13^	0.89	6.15 × 10^−5^	0.93	6.37 × 10^−6^	0.76	3.08 × 10^−3^
Brown	bta-miR-429	0.74	8.47 × 10^−12^	0.74	3.16 × 10^−3^	0.87	1.13 × 10^−4^	0.90	7.33 × 10^−5^
Brown	bta-miR-885	0.73	2.27 × 10^−11^	0.94	4.02 × 10^−6^	0.77	1.57 × 10^−3^	0.73	5.48 × 10^−3^
Brown	bta-miR-26b	0.72	5.57 × 10^−9^	0.74	2.98 × 10^−3^	0.87	1.32 × 10^−4^	0.72	6.31 × 10^−3^
Brown	bta-miR-30c	0.71	4.04 × 10^−13^	0.71	4.60 × 10^−3^	0.93	5.77 × 10^−6^	0.89	1.03 × 10^−4^
Brown	bta-let-7g	0.70	2.80 × 10^−8^	0.82	5.04 × 10^−4^	0.70	5.68 × 10^−3^	0.76	3.39 × 10^−3^
Brown	bta-miR-29b	0.64	7.43 × 10^−6^	0.68	7.75 × 10^−3^	0.64	1.26 × 10^−2^	0.68	1.03 × 10^−2^
Brown	bta-miR-328	0.63	1.02 × 10^−6^	0.63	1.39 × 10^−2^	0.81	6.48 × 10^−4^	0.64	1.67 × 10^−2^
Brown	bta-miR-32	0.63	2.25 × 10^−7^	0.63	1.43 × 10^−2^	0.78	1.51 × 10^−3^	0.78	2.35 × 10^−3^
Brown	bta-miR-107	0.61	4.32 × 10^−7^	0.61	1.78 × 10^−2^	0.77	1.79 × 10^−3^	0.77	2.66 × 10^−3^
Brown	bta-let-7a-3p	0.60	2.14 × 10^−8^	0.83	4.16 × 10^−4^	0.82	5.65 × 10^−4^	0.60	2.51 × 10^−2^
Turquoise	bta-miR-16b	0.85	4.24 × 10^−12^	0.86	1.93 × 10^−4^	0.86	1.85 × 10^−4^	0.85	4.94 × 10^−4^
Turquoise	bta-miR-130a	0.84	1.36 × 10^−15^	0.95	1.78 × 10^−6^	0.88	6.75 × 10^−5^	0.84	6.51 × 10^−4^
Turquoise	bta-miR-142-5p	0.84	2.35 × 10^−13^	0.88	7.90 × 10^−5^	0.89	4.54 × 10^−5^	0.84	6.66 × 10^−4^
Turquoise	bta-miR-218	0.81	2.47 × 10^−14^	0.85	2.40 × 10^−4^	0.81	7.09 × 10^−4^	0.95	3.45 × 10^−6^
Turquoise	bta-miR-142-3p	0.80	9.04 × 10^−13^	0.89	5.46 × 10^−5^	0.88	7.17 × 10^−5^	0.80	1.40 × 10^−3^
Turquoise	bta-miR-195	0.77	1.20 × 10^−12^	0.77	1.55 × 10^−3^	0.80	8.03 × 10^−4^	0.95	5.53 × 10^−6^
Turquoise	bta-miR-497	0.75	5.71 × 10^−13^	0.75	2.29 × 10^−3^	0.85	2.52 × 10^−4^	0.94	7.26 × 10^−6^
Turquoise	bta-miR-16a	0.74	2.61 × 10^−11^	0.82	5.21 × 10^−4^	0.74	2.91 × 10^−3^	0.92	3.83 × 10^−5^
Turquoise	bta-miR-19b	0.70	1.90 × 10^−8^	0.81	6.35 × 10^−4^	0.70	5.93 × 10^−3^	0.79	1.95 × 10^−3^
Turquoise	bta-miR-3613	0.68	4.50 × 10^−7^	0.76	2.12 × 10^−3^	0.68	7.00 × 10^−3^	0.72	6.31 × 10^−3^
Turquoise	bta-miR-455-3p	0.67	1.02 × 10^−6^	0.67	8.79 × 10^−3^	0.70	5.94 × 10^−3^	0.76	3.60 × 10^−3^
Turquoise	bta-miR-15a	0.66	1.15 × 10^−7^	0.85	2.03 × 10^−4^	0.67	8.90 × 10^−3^	0.66	1.30 × 10^−2^
Turquoise	bta-miR-424-5p	0.65	1.45 × 10^−8^	0.65	1.07 × 10^−2^	0.89	5.66 × 10^−5^	0.71	6.99 × 10^−3^
Turquoise	bta-miR-106b	0.64	4.81 × 10^−12^	0.89	5.64 × 10^−5^	0.64	1.18 × 10^−2^	0.93	2.05 × 10^−5^
Turquoise	bta-miR-155	0.64	2.68 × 10^−7^	0.83	4.77 × 10^−4^	0.69	6.30 × 10^−3^	0.64	1.68 × 10^−2^
Turquoise	bta-miR-455-5p	0.63	7.14 × 10^−7^	0.67	8.50 × 10^−3^	0.63	1.35 × 10^−2^	0.81	1.14 × 10^−3^
Turquoise	bta-miR-93	0.63	5.83 × 10^−10^	0.91	1.70 × 10^−5^	0.63	1.37 × 10^−2^	0.80	1.54 × 10^−3^
Turquoise	bta-miR-199a-5p	0.61	5.53 × 10^−6^	0.71	4.58 × 10^−3^	0.68	7.05 × 10^−3^	0.61	2.27 × 10^−2^
Turquoise	bta-miR-99a-3p	0.60	7.73 × 10^−7^	0.66	9.23 × 10^−3^	0.60	1.94 × 10^−2^	0.83	7.22 × 10^−4^

^1^ Eigengene−based connectivity (k.ME), a correlation coefficient of miRNA expression and the module eigengene value in all samples. k.ME is a measure of how the miRNA is correlated to module eigengene; ^2^ Correlation coefficient of miRNA expression and the module eigengene value in control samples; ^3^ Correlation coefficient of miRNA expression and the module eigengene value in linseed oil treatment; ^4^ Correlation coefficient of miRNA expression and the module eigengene value in safflower oil treatment.

**Table 3:** Enriched gene ontology (GO) terms for the blue, brown, and turquoise modules.

Module	* Term	GO ID	*p*-Value	** FDR
Blue	Vesicle docking	GO:0048278	8.02 × 10^−6^	1.53 × 10^−2^
Blue	Negative regulation of transcription from RNA Polymerase II promoter	GO: 0000122	2.49 × 10^−5^	2.38 × 10^−2^
Blue	Proteasome−mediated ubiquitin-dependent protein catabolic process	GO: 0043161	5.12 × 10^−5^	2.44 × 10^−2^
Blue	Protein dephosphorylation	GO: 0006470	4.44 × 10^−5^	2.44 × 10^−2^
Blue	Cell cycle arrest	GO: 0007050	1.02 × 10^−4^	3.69 × 10^−2^
Blue	RNA splicing	GO: 0008380	1.16 × 10^−4^	3.69 × 10^−2^
Brown	GDP binding	GO: 0019003	1.16 × 10^−9^	6.63 × 10^−7^
Brown	GTP binding	GO: 0005525	2.35 × 10^−8^	6.71 × 10^−6^
Brown	GTPase activity	GO: 0003924	9.34 × 10^−8^	1.78 × 10^−5^
Brown	RNA binding	GO: 0003723	4.05 × 10^−6^	5.78 × 10^−4^
Brown	Transforming growth factor β receptor signaling pathway	GO: 0007179	5.82 × 10^−7^	1.19 × 10^−3^
Brown	Protein serine/threonine kinase activity	GO: 0004674	1.72 × 10^−5^	1.40 × 10^−3^
Brown	Transforming growth factor β binding	GO: 0050431	1.52 × 10^−5^	1.40 × 10^−3^
Brown	Transforming growth factor β -activated receptor activity	GO: 0005024	1.48 × 10^−5^	1.40 × 10^−3^
Brown	Peptidyl-prolyl cis-trans isomerase activity	GO: 0003755	6.38 × 10^−5^	4.56 × 10^−3^
Brown	Ubiquitin protein ligase activity	GO: 0061630	8.32 × 10^−5^	5.28 × 10^−3^
Brown	Type I transforming growth factor β receptor binding	GO: 0034713	1.21 × 10^−4^	6.91 × 10^−3^
Brown	mRNA splicing, via spliceosome	GO: 0000398	7.53 × 10^−6^	7.70 × 10^−3^
Brown	Activin binding	GO: 0048185	4.78 × 10^−4^	2.48 × 10^−2^
Brown	Protein ubiquitination	GO: 0016567	4.28 × 10^−5^	2.92 × 10^−2^
Brown	Ubiquitin-protein transferase activity	GO: 0004842	7.29 × 10^−4^	3.47 × 10^−2^
Brown	Cell cycle arrest	GO: 0007050	9.44 × 10^−5^	4.83 × 10^−2^
Turquoise	GTP binding	GO: 0005525	6.32 × 10^−10^	4.13 × 10^−7^
Turquoise	Macroautophagy	GO: 0016236	2.44 × 10^−10^	5.31 × 10^−7^
Turquoise	Proteasome−mediated ubiquitin-dependent protein catabolic process	GO: 0043161	2.35 × 10^−9^	2.56 × 10^−6^
Turquoise	Membrane organization	GO: 0061024	4.22 × 10^−9^	3.06 × 10^−6^
Turquoise	RNA binding	GO: 0003723	4.15 × 10^−8^	9.27 × 10^−6^
Turquoise	GDP binding	GO: 0019003	4.26 × 10^−8^	9.27 × 10^−6^
Turquoise	Transcription coactivator activity	GO: 0003713	2.73 × 10^−7^	3.82 × 10^−5^
Turquoise	GTPase activity	GO: 0003924	2.93 × 10^−7^	3.82 × 10^−5^
Turquoise	Ubiquitin protein ligase activity	GO: 0061630	9.30 × 10^−7^	1.01 × 10^−4^
Turquoise	Ubiquitin protein ligase binding	GO: 0031625	1.10 × 10^−6^	1.03 × 10^−4^
Turquoise	Protein serine/threonine kinase activity	GO: 0004674	1.99 × 10^−6^	1.62 × 10^−4^
Turquoise	Protein K48-linked ubiquitination	GO: 0070936	5.71 × 10^−7^	3.10 × 10^−4^
Turquoise	Protein ubiquitination involved in ubiquitin-dependent protein catabolic process	GO: 0042787	9.25 × 10^−7^	4.02 × 10^−4^
Turquoise	Regulation of transcription from RNA polymerase II promoter	GO: 0006357	1.44 × 10^−6^	5.18 × 10^−4^
Turquoise	Protein deubiquitination	GO: 0016579	1.91 × 10^−6^	5.18 × 10^−4^
Turquoise	Nucleotide−excision repair, preincision complex assembly	GO: 0006294	1.77 × 10^−6^	5.18 × 10^−4^
Turquoise	Cadherin binding	GO: 0045296	1.10 × 10^−5^	7.99 × 10^−4^
Turquoise	Protein ubiquitination	GO: 0016567	5.19 × 10^−6^	1.25 × 10^−3^
Turquoise	Protein homodimerization activity	GO: 0042803	2.27 × 10^−5^	1.48 × 10^−3^
Turquoise	Protein polyubiquitination	GO: 0000209	7.21 × 10^−6^	1.57 × 10^−3^
Turquoise	Ubiquitin-protein transferase activity	GO: 0004842	3.22 × 10^−5^	1.91 × 10^−3^
Turquoise	Golgi organization	GO: 0007030	9.86 × 10^−6^	1.95 × 10^−3^
Turquoise	Transforming growth factor β receptor signaling pathway	GO: 0007179	1.13 × 10^−5^	2.04 × 10^−3^
Turquoise	G2/M transition of mitotic cell cycle	GO: 0000086	1.74 × 10^−5^	2.90 × 10^−3^
Turquoise	Negative regulation of apoptotic process	GO: 0043066	2.37 × 10^−5^	3.69 × 10^−3^
Turquoise	Positive regulation of apoptotic process	GO: 0043065	2.59 × 10^−5^	3.75 × 10^−3^
Turquoise	GABA receptor binding	GO: 0050811	7.94 × 10^−5^	4.32 × 10^−3^
Turquoise	Global genome nucleotide−excision repair	GO: 0070911	3.83 × 10^−5^	5.12 × 10^−3^
Turquoise	Stress-activated MAPK cascade	GO: 0051403	4.00 × 10^−5^	5.12 × 10^−3^
Turquoise	Positive regulation of ubiquitin-protein ligase activity involved in regulation of mitotic cell cycle transition	GO: 0051437	6.07 × 10^−5^	6.95 × 10^−3^
Turquoise	Protein K11-linked ubiquitination	GO: 0070979	5.94 × 10^−5^	6.95 × 10^−3^
Turquoise	Protein phosphorylation	GO: 0006468	6.40 × 10^−5^	6.96 × 10^−3^
Turquoise	Anaphase−promoting complex-dependent catabolic process	GO: 0031145	1.03 × 10^−4^	1.07 × 10^−2^
Turquoise	Post-translational protein modification	GO: 0043687	1.14 × 10^−4^	1.11 × 10^−2^
Turquoise	Virion assembly	GO: 0019068	1.18 × 10^−4^	1.11 × 10^−2^
Turquoise	Transforming growth factor β binding	GO: 0050431	2.84 × 10^−4^	1.32 × 10^−2^
Turquoise	Cadherin binding involved in cell-cell adhesion	GO: 0098641	2.84 × 10^−4^	1.32 × 10^−2^
Turquoise	Ligand-dependent nuclear receptor transcription coactivator activity	GO: 0030374	3.41 × 10^−4^	1.48 × 10^−2^
Turquoise	ER to Golgi vesicle−mediated transport	GO: 0006888	1.83 × 10^−4^	1.66 × 10^−2^
Turquoise	Protein kinase activity	GO: 0004672	4.47 × 10^−4^	1.69 × 10^−2^
Turquoise	R-SMAD binding	GO: 0070412	4.30 × 10^−4^	1.69 × 10^−2^
Turquoise	Ubiquitin conjugating enzyme binding	GO: 0031624	4.67 × 10^−4^	1.69 × 10^−2^
Turquoise	Ubiquitin-dependent protein catabolic process	GO: 0006511	2.03 × 10^−4^	1.70 × 10^−2^
Turquoise	COPII vesicle coating	GO: 0048208	1.96 × 10^−4^	1.70 × 10^−2^
Turquoise	Thiol-dependent ubiquitinyl hydrolase activity	GO: 0036459	5.21 × 10^−4^	1.79 × 10^−2^
Turquoise	Cellular response to DNA damage stimulus	GO: 0006974	2.41 × 10^−4^	1.87 × 10^−2^
Turquoise	Endocytosis	GO: 0006897	2.37 × 10^−4^	1.87 × 10^−2^
Turquoise	Negative regulation of sequence−specific DNA binding transcription factor activity	GO: 0043433	2.51 × 10^−4^	1.88 × 10^−2^
Turquoise	Regulation of signal transduction by p53 class mediator	GO: 1901796	2.80 × 10^−4^	1.94 × 10^−2^
Turquoise	Wnt signaling pathway, planar cell polarity pathway	GO: 0060071	2.72 × 10^−4^	1.94 × 10^−2^
Turquoise	Nucleotide−excision repair, DNA duplex unwinding	GO: 0000717	2.86 × 10^−4^	1.94 × 10^−2^
Turquoise	Transcription cofactor activity	GO: 0003712	6.37 × 10^−4^	2.08 × 10^−2^
Turquoise	Polynucleotide adenylyltransferase activity	GO: 0004652	6.74 × 10^−4^	2.09 × 10^−2^
Turquoise	Positive regulation of transcription from RNA polymerase II promoter	GO: 0045944	3.39 × 10^−4^	2.24 × 10^−2^
Turquoise	Activin binding	GO: 0048185	7.66 × 10^−4^	2.27 × 10^−2^
Turquoise	Androgen receptor signaling pathway	GO: 0030521	3.64 × 10^−4^	2.33 × 10^−2^
Turquoise	BMP signaling pathway	GO: 0030509	3.98 × 10^−4^	2.47 × 10^−2^
Turquoise	Guanyl-nucleotide exchange factor activity	GO: 0005085	9.54 × 10^−4^	2.71 × 10^−2^
Turquoise	Double−stranded DNA binding	GO: 0003690	1.10 × 10^−3^	2.98 × 10^−2^
Turquoise	Transcription from RNA polymerase II promoter	GO: 0006366	5.41 × 10^−4^	3.27 × 10^−2^
Turquoise	Retrograde transport, endosome to plasma membrane	GO: 1990126	6.11 × 10^−4^	3.59 × 10^−2^
Turquoise	Error-free translesion synthesis	GO: 0070987	6.32 × 10^−4^	3.62 × 10^−2^
Turquoise	Endosomal transport	GO: 0016197	6.73 × 10^−4^	3.66 × 10^−2^
Turquoise	GTP metabolic process	GO: 0046039	6.74 × 10^−4^	3.66 × 10^−2^
Turquoise	NIK/NF-γ B signaling	GO: 0038061	7.95 × 10^−4^	3.84 × 10^−2^
Turquoise	Negative regulation of transforming growth factor β receptor signaling pathway	GO: 0030512	7.75 × 10^−4^	3.84 × 10^−2^
Turquoise	Negative regulation of cell death	GO: 0060548	7.78 × 10^−4^	3.84 × 10^−2^
Turquoise	Nucleotide−excision repair, DNA incision, 5′-to lesion	GO: 0006296	7.78 × 10^−4^	3.84 × 10^−2^
Turquoise	Negative regulation of actin filament polymerization	GO: 0030837	7.66 × 10^−4^	3.84 × 10^−2^
Turquoise	Single−stranded DNA binding	GO: 0003697	1.61 × 10^−3^	4.19 × 10^−2^
Turquoise	G1/S transition of mitotic cell cycle	GO: 0000082	9.12 × 10^−4^	4.22 × 10^−2^
Turquoise	Negative regulation of ubiquitin-protein ligase activity involved in mitotic cell cycle	GO: 0051436	9.00 × 10^−4^	4.22 × 10^−2^
Turquoise	Cell cycle arrest	GO: 0007050	9.92 × 10^−4^	4.40 × 10^−2^
Turquoise	Nucleotide−excision repair, DNA incision	GO: 0033683	9.77 × 10^−4^	4.40 × 10^−2^
Turquoise	Positive regulation of I-γB kinase/NF-γB signaling	GO: 0043123	1.15 × 10^−3^	4.86 × 10^−2^
Turquoise	Regulation of small GTPase mediated signal transduction	GO: 0051056	1.22 × 10^−3^	4.86 × 10^−2^
Turquoise	Regulation of transcription from RNA polymerase II promoter in response to hypoxia	GO: 0061418	1.21 × 10^−3^	4.86 × 10^−2^
Turquoise	Wnt signaling pathway	GO: 0016055	1.28 × 10^−3^	4.86 × 10^−2^
Turquoise	Autophagy	GO: 0006914	1.31 × 10^−3^	4.86 × 10^−2^
Turquoise	Negative regulation of type I interferon production	GO: 0032480	1.32 × 10^−3^	4.86 × 10^−2^
Turquoise	Nucleotide−excision repair	GO: 0006289	1.32 × 10^−3^	4.86 × 10^−2^
Turquoise	Nucleotide−excision repair, preincision complex stabilization	GO: 0006293	1.25 × 10^−3^	4.86 × 10^−2^
Turquoise	Nucleotide−excision repair, DNA incision, 3′-to lesion	GO: 0006295	1.25 × 10^−3^	4.86 × 10^−2^
Turquoise	Alternative mRNA splicing, via spliceosome	GO: 0000380	1.31 × 10^−3^	4.86 × 10^−2^

* GABA: gamma-aminobutyric acid; MAPK: mitogen-activated protein kinase; R-SMAD: receptor-regulated SMADs; BMP: bone morphogenetic protein; NIK/NF-γB: NF- γB inducing kinase/nuclear factor-γB; ** Benjamini–Hochberg corrected *p*-values.

**Table 4:** Enriched KEGG pathways for the blue, brown, and turquoise modules.

Module	* Pathway	*p*-Value	** FDR
Blue	p53 signaling pathway	7.33 × 10^−6^	9.93 × 10^−4^
Blue	Cell cycle	5.05 × 10^−6^	9.93 × 10^−4^
Blue	Proteoglycans in cancer	6.12 × 10^−5^	5.53 × 10^−3^
Blue	HTLV-I infection	1.77 × 10^−4^	1.20 × 10^−2^
Blue	Epstein–Barr virus infection	3.27 × 10^−4^	1.78 × 10^−2^
Blue	ErbB signaling pathway	4.90 × 10^−4^	2.21 × 10^−2^
Blue	MAPK signaling pathway	6.61 × 10^−4^	2.38 × 10^−2^
Blue	Chronic myeloid leukemia	8.32 × 10^−4^	2.38 × 10^−2^
Blue	Huntington’s disease	8.52 × 10^−4^	2.38 × 10^−2^
Blue	Wnt signaling pathway	9.63 × 10^−4^	2.38 × 10^−2^
Blue	TGF-β signaling pathway	1.05 × 10^−3^	2.38 × 10^−2^
Blue	Hippo signaling pathway	1.02 × 10^−3^	2.38 × 10^−2^
Blue	Pathways in cancer	1.49 × 10^−3^	3.07 × 10^−2^
Blue	Protein processing in endoplasmic reticulum	1.59 × 10^−3^	3.07 × 10^−2^
Blue	FoxO signaling pathway	2.64 × 10^−3^	4.78 × 10^−2^
Brown	MAPK signaling pathway	2.73 × 10^−4^	3.40 × 10^−2^
Brown	TGF-β signaling pathway	1.83 × 10^−4^	3.40 × 10^−2^
Brown	Endocytosis	7.46 × 10^−4^	3.40 × 10^−2^
Brown	RNA degradation	6.58 × 10^−4^	3.40 × 10^−2^
Brown	p53 signaling pathway	6.43 × 10^−4^	3.40 × 10^−2^
Brown	Ubiquitin mediated proteolysis	7.30 × 10^−4^	3.40 × 10^−2^
Turquoise	Ubiquitin mediated proteolysis	2.15 × 10^−9^	5.81 × 10^−7^
Turquoise	Endocytosis	7.66 × 10^−8^	1.03 × 10^−5^
Turquoise	Protein processing in endoplasmic reticulum	8.37 × 10^−5^	7.53 × 10^−3^
Turquoise	p53 signaling pathway	1.69 × 10^−4^	1.14 × 10^−2^
Turquoise	Renal cell carcinoma	3.39 × 10^−4^	1.83 × 10^−2^
Turquoise	Focal adhesion	4.32 × 10^−4^	1.94 × 10^−2^
Turquoise	TGF-β signaling pathway	6.00 × 10^−4^	2.31 × 10^−2^
Turquoise	Regulation of autophagy	1.22 × 10^−3^	4.10 × 10^−2^
Turquoise	FoxO signaling pathway	1.83 × 10^−3^	4.48 × 10^−2^
Turquoise	Nucleotide excision repair	1.58 × 10^−3^	4.48 × 10^−2^
Turquoise	Cell cycle	1.70 × 10^−3^	4.48 × 10^−2^

* HPLV-I: human T-cell leukemia virus type I; FoxO: forkhead box O; MAPK: mitogen-activated protein kinase; TGF-β: transforming growth factor beta; ** Benjamini–Hochberg corrected *p*-values.

**Table 5:** Enriched transcription factors for the blue, brown, and turquoise modules.

Module	Transcription Factor	*p*-Value	* FDR
Blue	SMAD4	4.27 × 10^−10^	1.28 × 10^−7^
Blue	SP1	1.95 × 10^−9^	2.93 × 10^−7^
Blue	EGR1	3.50 × 10^−8^	3.50 × 10^−6^
Blue	ZBTB16	2.99 × 10^−5^	2.24 × 10^−3^
Blue	STAT3	1.52 × 10^−4^	7.59 × 10^−3^
Blue	CBFB	2.60 × 10^−4^	1.11 × 10^−2^
Blue	TP53	3.32 × 10^−4^	1.24 × 10^−2^
Blue	FOXJ1	5.05 × 10^−4^	1.52 × 10^−2^
Blue	NFYA	4.81 × 10^−4^	1.52 × 10^−2^
Blue	NRF1	5.86 × 10^−4^	1.57 × 10^−2^
Blue	LEF1	6.28 × 10^−4^	1.57 × 10^−2^
Blue	SRF	1.00 × 10^−3^	2.31 × 10^−2^
Blue	PPARG	1.33 × 10^−3^	2.85 × 10^−2^
Blue	E2F1	2.32 × 10^−3^	4.63 × 10^−2^
Brown	SP1	8.01 × 10^−11^	2.41 × 10^−8^
Brown	EGR1	1.30 × 10^−6^	1.30 × 10^−4^
Brown	SMAD4	1.12 × 10^−6^	1.30 × 10^−4^
Brown	TP53	1.90 × 10^−5^	1.43 × 10^−3^
Brown	E2F1	2.75 × 10^−5^	1.65 × 10^−3^
Brown	PLAU	5.86 × 10^−5^	2.94 × 10^−3^
Brown	NRF1	1.50 × 10^−4^	6.46 × 10^−3^
Brown	THRB	1.94 × 10^−4^	7.29 × 10^−3^
Brown	NRF1	2.20 × 10^−4^	7.35 × 10^−3^
Brown	ATF4	4.00 × 10^−4^	1.21 × 10^−2^
Brown	HIF1A	6.13 × 10^−4^	1.68 × 10^−2^
Brown	E2F6	9.46 × 10^−4^	2.37 × 10^−2^
Brown	CREM	1.63 × 10^−3^	3.76 × 10^−2^
Brown	STAT3	2.02 × 10^−3^	4.34 × 10^−2^
Turquoise	SMAD4	1.22 × 10^−13^	3.85 × 10^−11^
Turquoise	SP1	3.05 × 10^−11^	4.82 × 10^−9^
Turquoise	E2F1	1.40 × 10^−5^	8.86 × 10^−4^
Turquoise	SP4	2.76 × 10^−5^	1.46 × 10^−3^
Turquoise	THRB	6.70 × 10^−5^	3.02 × 10^−3^
Turquoise	NRF1	1.77 × 10^−4^	7.01 × 10^−3^
Turquoise	LEF1	2.24 × 10^−4^	7.86 × 10^−3^
Turquoise	MEF2A	5.02 × 10^−4^	1.59 × 10^−2^
Turquoise	CEBPD	5.97 × 10^−4^	1.66 × 10^−2^
Turquoise	GATA1	6.32 × 10^−4^	1.66 × 10^−2^
Turquoise	ATF4	7.62 × 10^−4^	1.76 × 10^−2^
Turquoise	ATF2	8.38 × 10^−4^	1.76 × 10^−2^
Turquoise	NFYA	8.29 × 10^−4^	1.76 × 10^−2^
Turquoise	IRF8	1.17 × 10^−3^	2.32 × 10^−2^
Turquoise	NFAT2	1.39 × 10^−3^	2.45 × 10^−2^
Turquoise	STAT3	1.39 × 10^−3^	2.45 × 10^−2^
Turquoise	ELK4	2.03 × 10^−3^	3.21 × 10^−2^
Turquoise	TCFAP2A	1.97 × 10^−3^	3.21 × 10^−2^
Turquoise	PITX1	2.37 × 10^−3^	3.56 × 10^−2^
Turquoise	E2F6	2.76 × 10^−3^	3.97 × 10^−2^
Turquoise	SPI1	2.92 × 10^−3^	4.02 × 10^−2^
Turquoise	GTF2I	3.26 × 10^−3^	4.02 × 10^−2^
Turquoise	MAX	3.24 × 10^−3^	4.02 × 10^−2^
Turquoise	TEAD4	3.31 × 10^−3^	4.02 × 10^−2^
Turquoise	HNF1A	3.87 × 10^−3^	4.40 × 10^−2^
Turquoise	HINFP	4.13 × 10^−3^	4.50 × 10^−2^

* Benjamini–Hochberg corrected *p*-values.

**Table 6:** Correlation of significant miRNA–mRNA pairs with phenotypes.

Module	miRNA	Gene Symbol	^1^ Context ++ Score Percentile	^2^ Cor. Mir. Gene	^3^ FDR. Cor. Mir. Gene	Trait	^4^ Cor. Trait Mir.	^5^ FDR. Cor. Trait Mir.	^6^ Cor. Gene. Trait	^7^ FDR. Cor. Gene. Trait
Blue	bta-let-7a-5p	STX3	97	−0.428	0.022	Protein percentage	0.484	0.010	−0.438	0.023
Blue	bta-let-7b	APBB3	98	−0.394	0.037	Protein yield	−0.400	0.041	0.620	<0.001
Blue	bta-let-7b	C14orf28	97	−0.485	0.008	Milk yield	−0.509	0.006	0.424	0.029
Blue	bta-let-7b	MXD1	97	−0.457	0.014	Protein yield	−0.400	0.041	0.446	0.020
Blue	bta-let-7b	PPP1R15B	96	−0.420	0.025	Protein yield	−0.400	0.041	0.609	0.001
Blue	bta-let-7b	QARS	98	−0.386	0.041	Milk yield	−0.509	0.006	0.491	0.009
Blue	bta-let-7b	SLC20A1	96	−0.391	0.039	Milk yield	−0.509	0.006	0.502	0.007
Blue	bta-let-7b	STX3	97	−0.464	0.012	Protein yield	−0.400	0.041	0.531	0.004
Blue	bta-let-7b	THTPA	98	−0.431	0.021	Protein yield	−0.400	0.041	0.409	0.036
Blue	bta-let-7b	TP53	97	−0.413	0.028	Protein yield	−0.400	0.041	0.459	0.016
Blue	bta-miR-183	CTDSP1	98	−0.433	0.020	Protein yield	−0.452	0.018	0.443	0.021
Blue	bta-miR-183	DGCR2	99	−0.516	0.005	Protein yield	−0.452	0.018	0.442	0.021
Blue	bta-miR-183	HLTF	98	−0.428	0.022	Protein yield	−0.452	0.018	0.502	0.007
Blue	bta-miR-183	HNRNPA1	96	−0.479	0.009	Milk yield	−0.599	0.001	0.414	0.034
Blue	bta-miR-183	ICA1	99	−0.443	0.017	Protein yield	−0.452	0.018	0.548	0.003
Blue	bta-miR-183	ILF2	96	−0.373	0.050	C17:0	0.414	0.033	−0.400	0.041
Blue	bta-miR-183	MAFF	97	−0.427	0.022	Milk yield	−0.599	0.001	0.467	0.014
Blue	bta-miR-183	MGME1	98	−0.456	0.014	Milk yield	−0.599	0.001	0.399	0.042
Blue	bta-miR-183	MTA1	99	−0.475	0.010	Protein yield	−0.452	0.018	0.415	0.033
Blue	bta-miR-183	PPP2R5C	95	−0.446	0.017	Milk yield	−0.599	0.001	0.433	0.025
Blue	bta-miR-183	RHBDD2	95	−0.538	0.003	Protein percentage	0.547	0.003	−0.411	0.035
Blue	bta-miR-183	RHPN2	99	−0.407	0.031	Milk yield	−0.599	0.001	0.433	0.025
Blue	bta-miR-183	SESN1	97	−0.375	0.048	Protein yield	−0.452	0.018	0.396	0.043
Blue	bta-miR-183	SFT2D1	97	−0.500	0.006	C17:0	0.414	0.033	−0.425	0.028
Blue	bta-miR-183	SPRY2	99	−0.418	0.026	Protein yield	−0.452	0.018	0.488	0.009
Blue	bta-miR-183	SRSF2	98	−0.473	0.010	Protein yield	−0.452	0.018	0.617	0.001
Blue	bta-miR-183	UTP6	96	−0.432	0.021	protein yield	−0.452	0.018	0.545	0.003
Blue	bta-miR-183	ZFAND5	99	−0.427	0.022	protein yield	−0.452	0.018	0.430	0.026
Blue	bta-miR-2284b	ACVR1	96	−0.419	0.025	Milk yield	−0.442	0.021	0.416	0.032
Blue	bta-miR-2284b	ARL15	97	−0.443	0.017	protein yield	−0.429	0.026	0.427	0.027
Blue	bta-miR-2284b	CCNT2	96	−0.398	0.035	protein yield	−0.429	0.026	0.439	0.023
Blue	bta-miR-2284b	CLIC2	99	−0.416	0.027	protein yield	−0.429	0.026	0.610	0.001
Blue	bta-miR-2284b	ERG	95	−0.448	0.016	protein yield	−0.429	0.026	0.393	0.045
Blue	bta-miR-2284b	FAM114A1	96	−0.425	0.023	Milk yield	−0.442	0.021	0.459	0.016
Blue	bta-miR-2284b	FAM8A1	95	−0.386	0.042	protein yield	−0.429	0.026	0.414	0.033
Blue	bta-miR-2284b	FAR1	95	−0.387	0.041	Milk yield	−0.442	0.021	0.399	0.042
Blue	bta-miR-2284b	IVNS1ABP	95	−0.404	0.032	protein yield	−0.429	0.026	0.509	0.006
Blue	bta-miR-2284b	LBR	96	−0.510	0.005	protein yield	−0.429	0.026	0.462	0.015
Blue	bta-miR-2284b	LIMA1	97	−0.446	0.017	protein yield	−0.429	0.026	0.403	0.039
Blue	bta-miR-2284b	NRROS	96	−0.406	0.031	Milk yield	−0.442	0.021	0.480	0.011
Blue	bta-miR-2284b	POLR2A	99	−0.415	0.027	protein yield	−0.429	0.026	0.667	0.000
Blue	bta-miR-2284b	RRN3	96	−0.377	0.047	protein yield	−0.429	0.026	0.501	0.007
Blue	bta-miR-2284b	SETD2	97	−0.399	0.035	protein yield	−0.429	0.026	0.438	0.023
Blue	bta-miR-2284b	SLC38A2	95	−0.417	0.026	Milk yield	−0.442	0.021	0.526	0.004
Blue	bta-miR-2284b	THAP2	96	−0.385	0.042	protein yield	−0.429	0.026	0.445	0.020
Blue	bta-miR-2284b	UBE4A	96	−0.414	0.027	protein yield	−0.429	0.026	0.490	0.009
Blue	bta-miR-2284b	ZDHHC17	95	−0.384	0.043	protein yield	−0.429	0.026	0.426	0.028
Blue	bta-miR-2284b	ZNF175	97	−0.407	0.031	Milk yield	−0.442	0.021	0.433	0.025
Blue	bta-miR-23b-3p	RBM4B	95	−0.463	0.012	Milk yield	−0.390	0.047	0.549	0.003
Blue	bta-miR-30d	CAMK2D	95	−0.384	0.042	protein percentage	0.459	0.016	−0.433	0.025
Blue	bta-miR-409a	ALG13	99	−0.490	0.008	Milk yield	−0.553	0.002	0.462	0.015
Blue	bta-miR-409a	GALNT5	96	−0.482	0.009	Protein percentage	0.641	0.000	−0.616	0.001
Blue	bta-miR-409a	RPL11	98	−0.484	0.009	Fat percentage	0.387	0.049	−0.447	0.020
Blue	bta-miR-409a	TMEM159	99	−0.410	0.029	Fat percentage	0.387	0.049	−0.396	0.044
Blue	bta-miR-409a	TRA2B	97	−0.382	0.044	Milk yield	−0.553	0.002	0.598	0.001
Blue	bta-miR-6522	FAM107B	95	−0.384	0.043	Milk yield	−0.441	0.022	0.554	0.002
Blue	bta-miR-6522	ZNF623	95	−0.376	0.048	Milk yield	−0.441	0.022	0.550	0.003
Blue	bta-miR-96	CHST1	98	−0.373	0.050	Milk yield	−0.429	0.026	0.485	0.010
Blue	bta-miR-96	EIF5	96	−0.416	0.027	Milk yield	−0.429	0.026	0.494	0.009
Blue	bta-miR-96	FARP1	97	−0.466	0.012	Milk yield	−0.429	0.026	0.599	0.001
Blue	bta-miR-96	GRHL2	95	−0.398	0.035	Milk yield	−0.429	0.026	0.481	0.011
Blue	bta-miR-96	LONP2	97	−0.439	0.019	Fat percentage	0.613	0.001	−0.415	0.033
Blue	bta-miR-96	PRKAR1A	97	−0.375	0.049	Milk yield	−0.429	0.026	0.442	0.022
Blue	bta-miR-96	SPIN1	95	−0.392	0.038	Milk yield	−0.429	0.026	0.533	0.004
Blue	bta-miR-96	SPROT	97	−0.410	0.029	Milk yield	−0.429	0.026	0.535	0.004
Blue	bta-miR-96	TP53	95	−0.440	0.018	Milk yield	−0.429	0.026	0.427	0.027
Blue	bta-miR-96	TRIB3	97	−0.397	0.035	Fat percentage	0.613	0.001	−0.550	0.003
Blue	bta-miR-96	ZCCHC3	99	−0.453	0.015	Milk yield	−0.429	0.026	0.567	0.002
Brown	bta-miR-484	CPPED1	95	−0.413	0.028	C22:6n3	−0.402	0.040	0.600	0.001
Brown	bta-miR-484	DOLPP1	96	−0.393	0.037	C16:0	0.394	0.045	−0.396	0.043
Brown	bta-miR-484	EIF1AD	95	−0.470	0.011	Fat percentage	0.421	0.030	−0.432	0.025
Brown	bta-miR-484	LY6E	97	−0.420	0.025	C16:0	0.394	0.045	−0.394	0.045
Brown	bta-miR-484	NUDT16	96	−0.390	0.039	Fat percentage	0.421	0.030	−0.456	0.017
Brown	bta-miR-484	QDPR	99	−0.391	0.039	C16:0	0.394	0.045	−0.596	0.001
Turquoise	bta-miR-130a	SBSPON	96	−0.529	0.004	Protein percentage	−0.486	0.010	0.626	< 0.001
Turquoise	bta-miR-455-5p	HPGD	99	−0.384	0.043	Protein yield	0.491	0.009	−0.492	0.009

^1^ Context++ score percentile from TargetScan prediction; ^2^ Correlation coefficient between miRNA and gene; ^3^ FDR (Benjamini–Hochberg corrected *p*-values) for Pearson correlation between miRNA and gene; ^4^ Correlation coefficient between miRNA and trait; ^5^ FDR for Pearson correlation between miRNA and trait; ^6^ Correlation coefficient between gene and trait; ^7^ FDR for Pearson correlation between gene and trait.

## Supplementary Materials

Supplementary materials can be found at http://www.mdpi.com/1422-0067/19/9/2500/s1. Table S1a–g: Predicted target genes (mRNAs) for miRNA members of the (a) blue, (c) brown, and (e) turquoise modules. Predicted filtered target genes for miRNA members of the (b) blue, (d) brown and (f) turquoise modules (these are predicted target mRNAs of the miRNAs that were present in the mRNA transcriptome data of the same animals and negatively correlated with any of the miRNAs in the blue, brown, and turquoise modules) (predicted target mRNAs that were not present in the mRNA transcriptome of the same data were not further used). (g) Specific mRNA-miRNA pairs in the blue, brown, and turquoise modules (each pair contains a miRNA that is negatively correlated with its target gene. Figure S1: The dynamic cut tree obtained from weighted co-expression network analyses.
