# Supplementary figures and images for: Co-Expression Network Analysis Identifies miRNA–mRNA Networks Potentially Regulating Milk Traits and Blood Metabolites
Source: Int J Mol Sci. 2018 Aug 24;19(9):2500. doi: 10.3390/ijms19092500 (PMC6164576; doi:10.3390/ijms19092500)

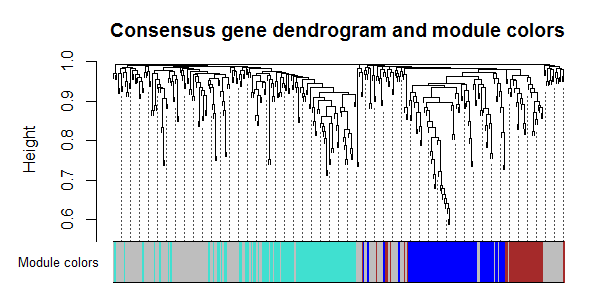

Supplement: Supplementary file 1 [file ijms-19-02500-s001.zip › figure S1.Consensus gene dendrogram and module color.tiff]
